# Zika virus infection leads to hormone deficiencies of the hypothalamic-pituitary-gonadal axis and diminished fertility in mice

**DOI:** 10.1128/jvi.01006-23

**Published:** 2023-09-21

**Authors:** Li-Bo Liu, Wei Yang, Jia-Tong Chang, Dong-Ying Fan, Yan-Hua Wu, Pei-Gang Wang, Jing An

**Affiliations:** 1 Department of Microbiology, School of Basic Medical Sciences, Capital Medical University, Beijing, China; 2 Department of Neurosurgery, Capital Medical University Sanbo Brain Hospital, Beijing, China; 3 Center of Epilepsy, Beijing Institute for Brain Disorders, Beijing, China; University of North Carolina at Chapel Hill, Chapel Hill, North Carolina, USA

**Keywords:** Zika virus, hypothalamus, hormone deficiencies, developmental delays, testis, fertility

## Abstract

Zika virus (ZIKV) infection in pregnant women during the first trimester can cause congenital malformations including microcephaly, which has focused global attention on this emerging pathogen. By contrast, ZIKV infection in pregnant women during the third trimester can cause neurodevelopmental delays and cryptorchidism in newborns without microcephaly. Here, we evaluated the long-term fertility consequences of ZIKV infection in male suckling mice, which is equivalent to congenital ZIKV infection in children, from multiple perspectives including the hypothalamic-pituitary-gonadal axis, testis, sperm, and social behaviors. We observed the persistence of ZIKV in the hypothalamus of mice, which caused long-term hormone deficiencies of the hypothalamic-pituitary-gonadal axis. Hormone deficiencies led to limited delays in testicular development, a decrease in sperm quality and quantity, and abnormal social abilities. These damages could progress to diminished fertility in male mice, as judged by lower pregnancy rate, prolonged time to delivery, and decreased offspring quality. Currently, the extent to which these observations in mice translate to humans remains unclear, but longitudinal studies of hormone levels and reproductive health in ZIKV-exposed children seem warranted.

**IMPORTANCE:**

Zika virus (ZIKV) infection in pregnant women during the third trimester can cause neurodevelopmental delays and cryptorchidism in children without microcephaly. However, the consequences of congenital ZIKV infection on fertility in these children remain unclear. Here, using an immunocompetent mouse model, we reveal that congenital ZIKV infection can cause hormonal disorders of the hypothalamic-pituitary-gonadal axis, leading to reduced fertility and decreased sexual preference. Our study has for the first time linked the hypothalamus to the reproductive system and social behaviors after ZIKV infection. Although the extent to which these observations in mice translate to humans remains unclear, these findings did suggest that the reproductive health and hormone levels of ZIKV-exposed children should receive more attention to improve their living quality.

## INTRODUCTION

Zika virus (ZIKV), a member of mosquito-borne flaviviruses, is primarily transmitted by Aedes mosquitoes (1); however, sexual transmission and vertical transmission of ZIKV have been reported (2). ZIKV was first discovered in the Zika jungle of Uganda, Africa in 1947 (3), and emerged as a public health concern during the ZIKV epidemic from the Asia-Pacific region to the Americas in 2015–2017 (4, 5). ZIKV affected millions of people in 87 countries and territories, caused thousands of microcephaly cases, and aggravated the disease burden on countless families (6, 7).

ZIKV infection in pregnant women during the first trimester can cause congenital malformations including microcephaly (8 – 10). By contrast, ZIKV infection of pregnant women during the third trimester receives little attention because their newborns did not exhibit apparent congenital malformations (11). Epidemiological investigations indicated that these newborns also had a variety of developmental complications (12 – 17). In Brazil, an investigation of 115 three-month-old neonates showed that 56 ZIKV-exposed neonates had significantly lower weight and body length than 59 non-exposed neonates (15). Recent studies also revealed that the majority of ZIKV-exposed children had developmental delays in cognition, language, motor, and adaptation, especially social-emotional abilities from 7 to 32 months old (12 – 14). In 2019, an investigation of 22 male children exposed to ZIKV showed that 8 of them had cryptorchidism (17). These studies suggested that ZIKV infection can cause various developmental delays in newborns, but the long-term consequences of neonatal ZIKV infection on their reproductive health remain unclear.

In our previous study, using ZIKV-infected suckling mice, we observed that ZIKV infection in the hypothalamus caused hormone deficiencies along the hypothalamic-pituitary-thyroid axis, resulting in irreversible growth delay and memory impairment (18). As the superior center of the neuroendocrine system, the hypothalamus also plays an important role in fertility by regulating the hypothalamic-pituitary-gonadal (HPG) axis. Thus, whether ZIKV infection affects reproductive development via the HPG axis remains unclear. In the current study, using ZIKV-infected male suckling mice, we observed that ZIKV infection caused long-term hormone deficiencies of the HPG axis, accompanied by phenotypes associated with reproductive endocrine disorders in mice. The impacts of ZIKV infection on the reproductive endocrine system and fertility persisted up to the adulthood of mice. Thus, our results provided clues to appreciate the potential impacts of ZIKV infection on the reproductive health and hormone levels of children in the epidemic area.

## RESULTS

### Postnatal ZIKV infection causes diminished fertility in male mice

**Fig 1:**
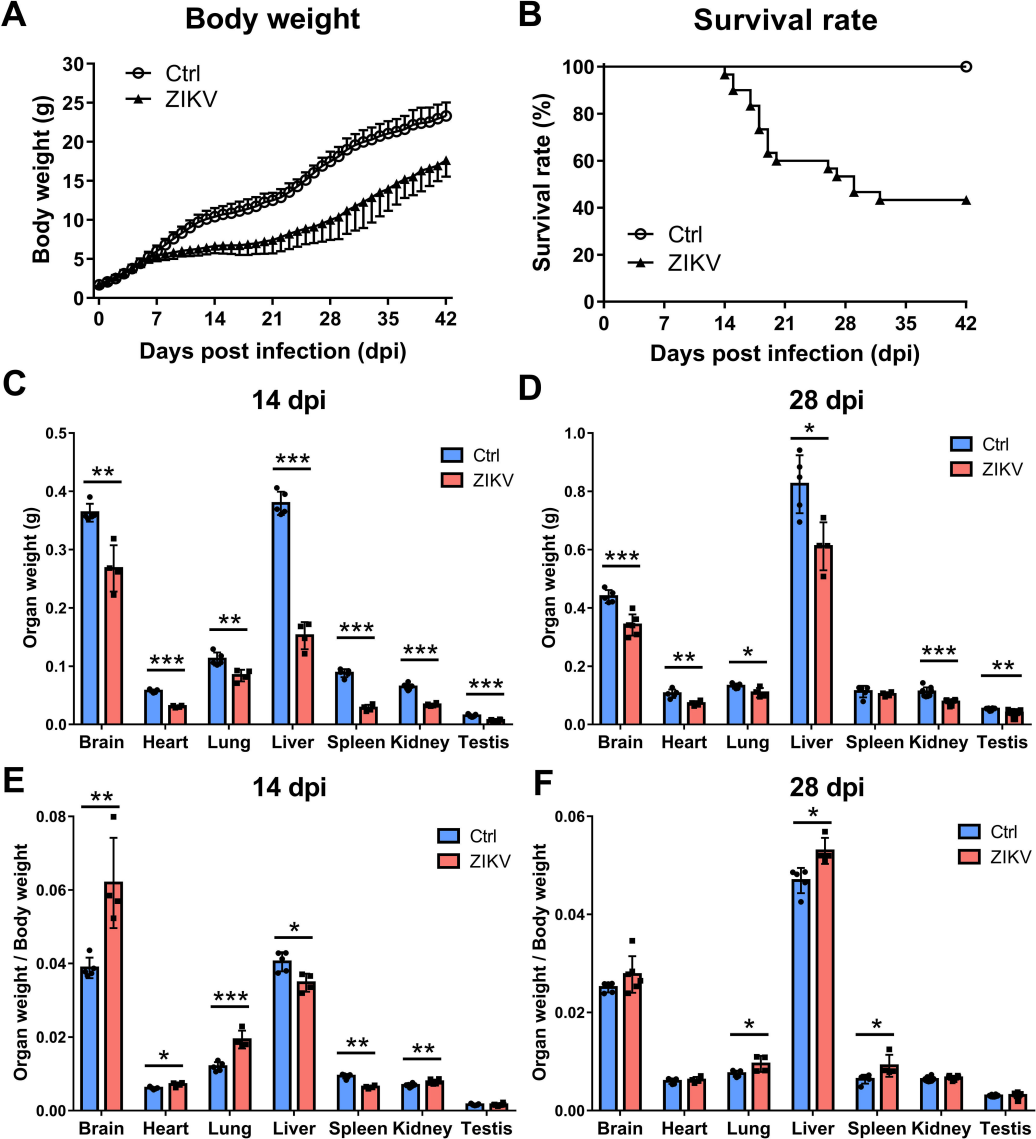
Body weight, survival rate, organ weight, and organ coefficient of neonatal ZIKV-infected mice. BALB/c male mice at postnatal day 2 were intracerebrally inoculated with 100 plaque-forming units of ZIKV or phosphate-buffered saline. (**A**) Body weight and (**B**) survival rate were inspected daily for 42 days after infection (*n* = 11 for the Ctrl group; *n* = 30 for the ZIKV group). (**C and D**) Weight of brain, heart, lung, liver, spleen, kidney, and testis in ZIKV-infected and control mice (*n* = 4 to 5 for each time point) at (**C**) 14 dpi and (**D**) 28 dpi. (**E and F**) Organ coefficients of brain, heart, lung, liver, spleen, kidney, and testis in ZIKV-infected and control mice at (**E**) 14 dpi and (**F**) 28 dpi. (*P < 0.05; **P < 0.01; ***P < 0.001; Student’s *t*-test).

Male suckling mice were intracerebrally inoculated with ZIKV at postnatal day 2 (P2), which is equivalent to the neonatal infection during the third trimester (19, 20). Compared with control mice, ZIKV-infected male mice had significantly lower body weight from 7 to 56 days post-infection (dpi) (Fig. 1A), and 43.3% of them survived more than 6 weeks (Fig. 1B). In addition, ZIKV-infected mice had remarkably lower weights of brain, heart, lung, liver, kidney, and testes (Fig. 1C through F).

**Fig 2:**
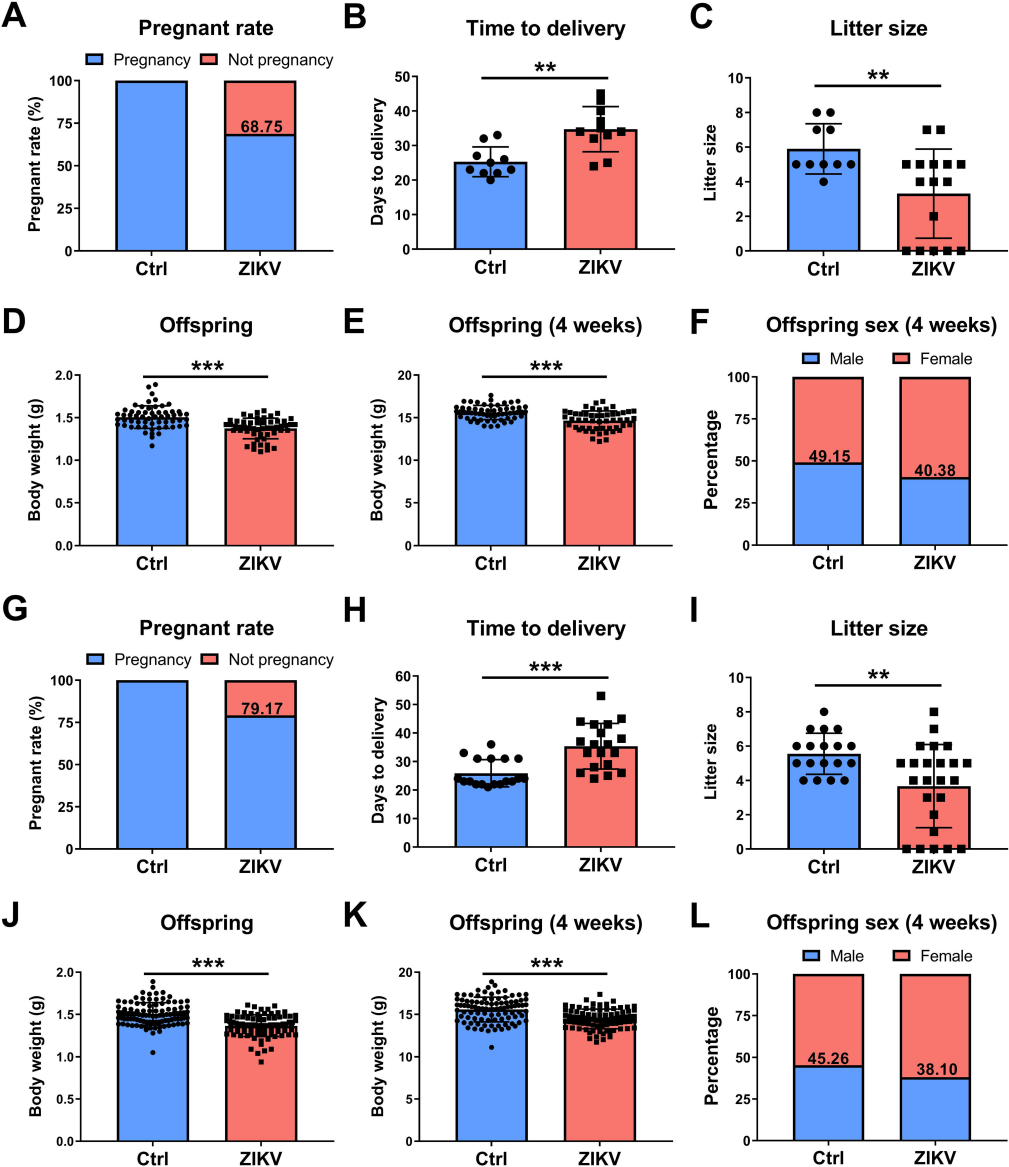
Mating experiments of neonatal ZIKV-infected mice at 6 and 8 weeks old. 6-week-old and 8-week-old ZIKV-infected and control male mice were mated with healthy age-matched female mice in a ratio of 1:2. (**A**) Pregnant rate, (**B**) the time from being co-caged to delivery of female mice, (**C**) the litter size, (**D**) birth weight, (**E**) body weight at 4 weeks, and (**F**) the sex ratio of offspring mice were recorded in mating experiments of 6-week-old mice (*n* = 5 for the Ctrl group; *n* = 8 for the ZIKV group). (**G**) Pregnant rate, (**H**) the time from being co-caged to delivery of female mice, (**I**) the litter size, (**J**) birth weight, (**K**) body weight at 4 weeks, and (**L**) the sex ratio of offspring mice were recorded in mating experiments of 8-week-old mice (*n* = 9 for the Ctrl group; *n* = 12 for the ZIKV group). (**P < 0.01; ***P < 0.001; Mann-Whitney U test for the litter size; Student’s *t*-test for others).

To determine whether postnatal ZIKV infection affects fertility in male mice, ZIKV-infected male mice were mated with healthy age-matched female mice when they were 6 or 8 weeks old (Fig. 2A through L). The pregnancy rate, the time from being co-caged to delivery, and the litter size were recorded. Although 68.75% (11/16, 6 weeks old) or 79% (19/24, 8 weeks old) of female mice in cages with ZIKV-infected male mice were able to become pregnant (Fig. 2A and G), they had significantly longer time (up to 53 days) to delivery (Fig. 2B and H). ZIKV-infected mice also had fewer litter sizes than control mice (Fig. 2C and I). In addition, we observed noticeably lower birth weight, lower 4-week weight, and more females in offspring of ZIKV-infected mice (Fig. 2D through F and J through L). These results indicated decreased fertility in postnatal ZIKV-infected mice in adulthood.

### Limited developmental delays in testes of ZIKV-infected mice

**Fig 3:**
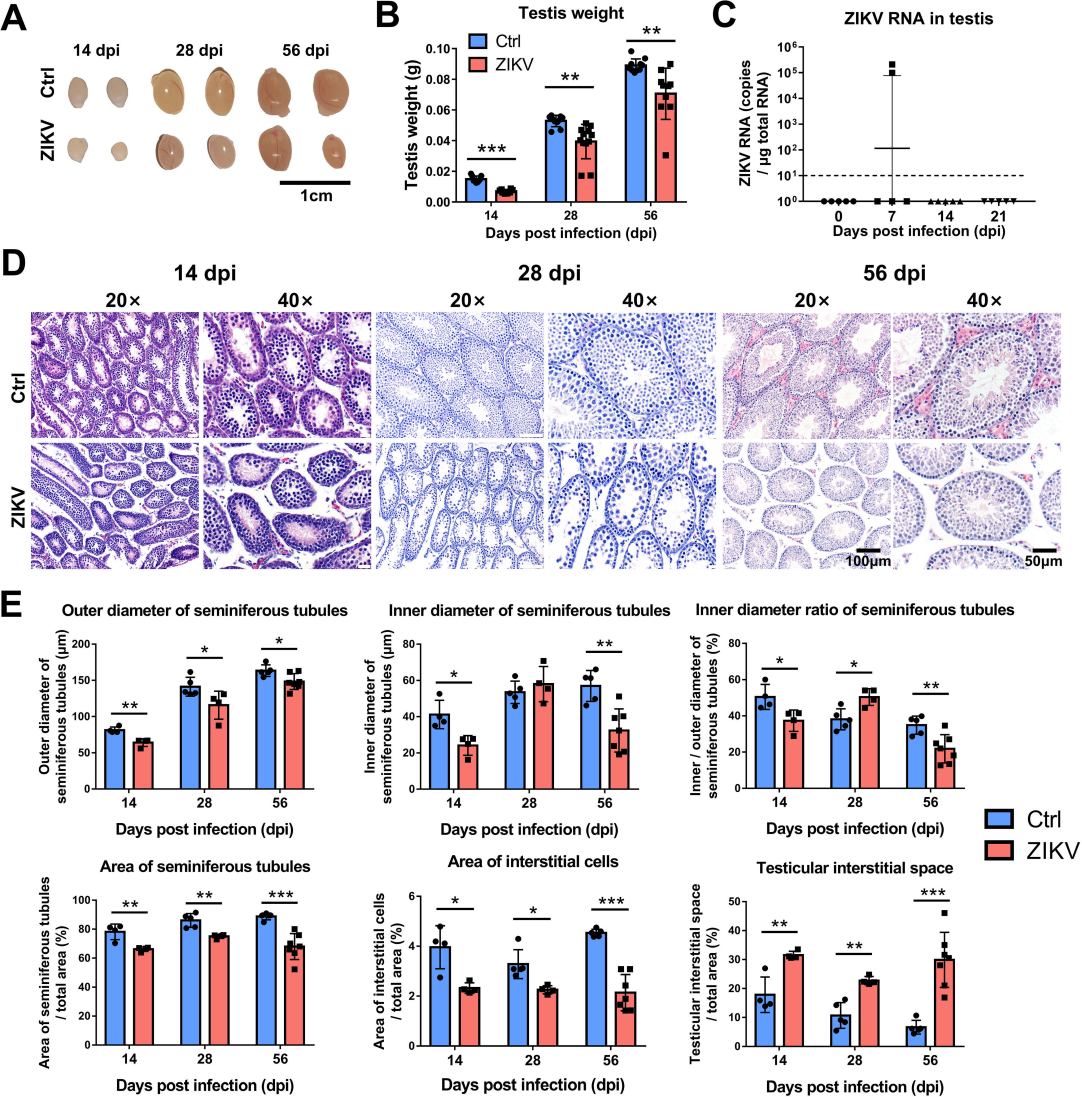
Effects of neonatal ZIKV infection on mice testes. Testes were collected from ZIKV-infected and control mice at 14, 28, and 56 dpi (*n* = 5 to 6 male mice for each time point). (**A**) Representative images of testis from ZIKV-infected and control mice at 14, 28, and 56 dpi; scale bar = 1 cm. (**B**) Testis weight from ZIKV-infected and control mice at 14, 28, and 56 dpi (**P < 0.01; ***P < 0.001; Student’s *t*-test). (**C**) Testes collected were analyzed for viral RNA by RT-qPCR. The dashed line indicates the detection limit. (**D**) Hematoxylin and eosin (HE) staining of testes sections collected from ZIKV-infected and control mice at 20× (left) and 40× (right) magnification. (**E**) Outer diameter, inner diameter, diameter ratio, area of seminiferous tubules, area of interstitial cells, and interstitial space were measured from HE staining of testes sections (*P < 0.05; **P < 0.01; ***P < 0.001; Student’s *t*-test).

The testis is a key organ for spermatogenesis. To determine whether the diminished fertility results from ZIKV-induced damage to testes, we evaluated pathological changes in mouse testes. We observed smaller testes in ZIKV-infected mice than in control mice at 14, 28, and 56 dpi (Fig. 3A and B), while the testis organ coefficient was similar in the two groups (Fig. 1E and F). ZIKV RNA was detected in part (2/5) of mouse testes only at 7 dpi (Fig. 3C). These results indicated that the testicular development was coordinated with the overall development in ZIKV-infected mice. A few mice had transient testicular infections, which was similar to the detection rate of ZIKV in men’s semen, as reported (21).

Hematoxylin and eosin (HE) staining showed atrophy of seminiferous tubules in the testes of ZIKV-infected mice, as judged by irregular tubule morphology, reduced outer diameter, and extended interstitium between seminiferous tubules (Fig. 3D and E). Over time, seminiferous tubules of ZIKV-infected mouse testes became more regular morphology, and their spermatogenic cells increased (Fig. 3D and E). There was no obvious inflammatory cell infiltration and loss of integrity of the blood-testis-barrier (BTB) in the testis of ZIKV-infected mice. These results indicated that testes of postnatal ZIKV-infected mice were not severely damaged, albeit developmental delays, which is distinct from severe destruction observed in ZIKV-infected Ifnar1^-/-^ mice (22, 23) or C57BL/6 mice treated with anti-Ifnar1 blocking monoclonal antibody (24).

**Fig 4:**
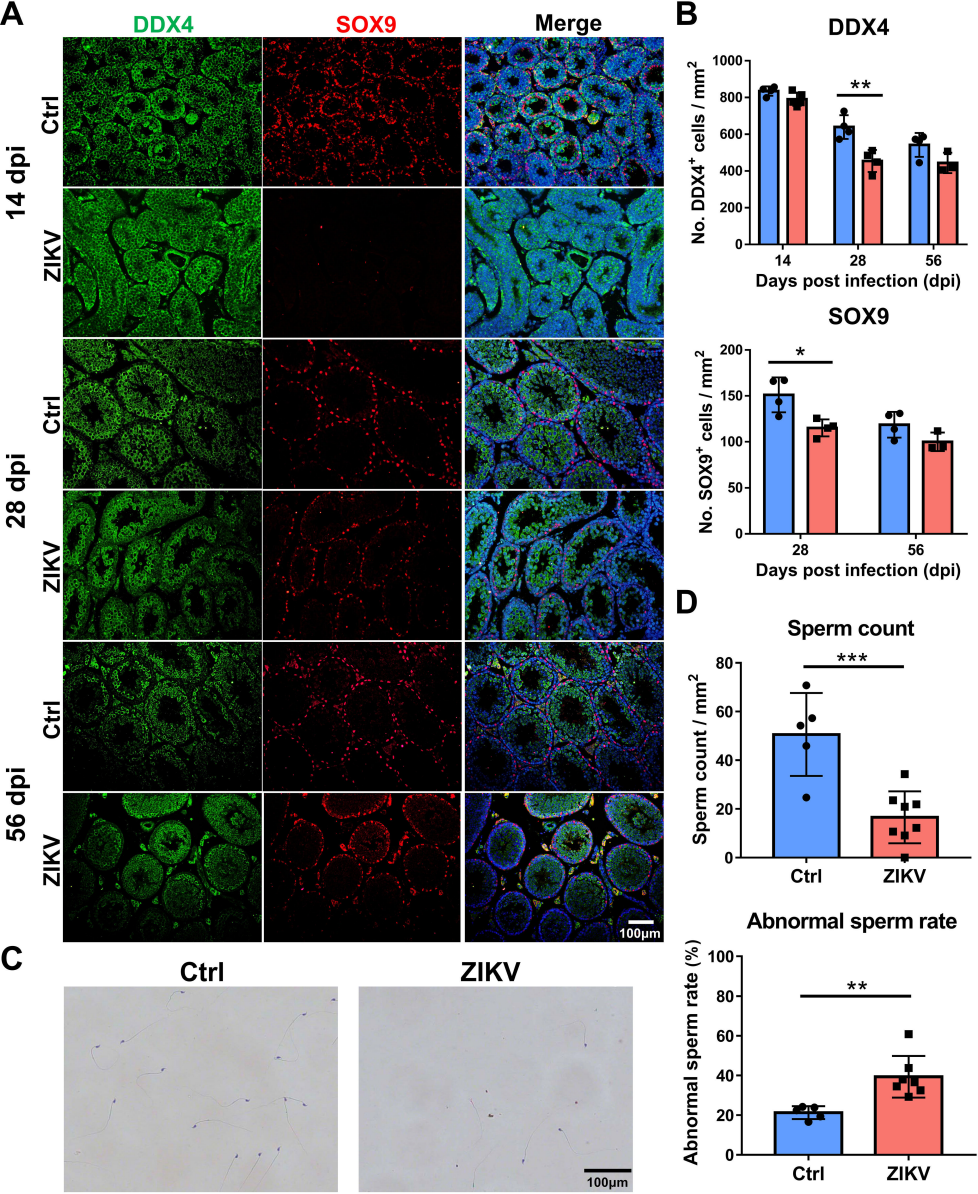
Effects of neonatal ZIKV infection on testes and sperm of mice. (**A**) Immunofluorescence (IF) staining of testes sections collected from ZIKV-infected and control mice with antibodies to DDX4 (spermatogenic cells) and SOX9 (Sertoli cells). Scale bars = 100 µm. (**B**) The number of DDX4^+^ cells and SOX9^+^ cells was counted from IF staining of testes sections (*P < 0.05; **P < 0.01; Student’s *t*-test). (**C**) Papanicolaou staining of sperm smears harvested from the epididymis of ZIKV-infected and control mice at 56 dpi. Scale bars = 100 µm. (**D**) Sperm count and abnormal sperm rate were counted from Papanicolaou staining of sperm smears (**P < 0.01; ***P < 0.001; Student’s *t*-test).

Immunofluorescence staining for DDX4 (a spermatogenic cell marker) and SOX9 (a Sertoli cell marker) was performed to examine the number and distribution of spermatogenic cells and Sertoli cells in the testes. At 14 dpi, ZIKV-infected and control mice had similar numbers of DDX4^+^ cells, while SOX9^+^ cells were not observed in ZIKV-infected mice (Fig. 4A and B). Subsequently, ZIKV-infected mice had significantly fewer DDX4^+^ and SOX9^+^ cells than control mice at 28 dpi; however, there was no statistical difference between the two groups at 56 dpi (Fig. 4A and B). These results indicated reversible developmental delays of spermatogenic cells and Sertoli cells in the testes of ZIKV-infected mice.

Papanicolaou staining for sperm smears harvested from epididymis at 56 dpi showed significantly less sperm in ZIKV-infected mice compared with control mice (Fig. 4C and D). ZIKV-infected mice had a higher rate of abnormal sperm (e.g., capitulum, headless, angular neck, double tail, and short tail) (Fig. 4D). These results indicated the decreased quantity and quality of sperm in ZIKV-infected mice.

Taken together, testes of postnatal ZIKV-infected mice had limited developmental delays, which was distinct from severe destruction observed in testes of ZIKV-infected C57BL/6 mice treated with anti-Ifnar1 blocking monoclonal antibody (24) or ZIKV-infected Ifnar1^-/-^ mice (22, 23). Thus, the longer time to delivery, diminished fertility, and decreased offspring quality in ZIKV-infected mice were most likely due to the decrease in sperm quantity and quality, rather than the damage of ZIKV to the testes.

### Reduced sociability and preference for females in ZIKV-infected mice

**Fig 5:**
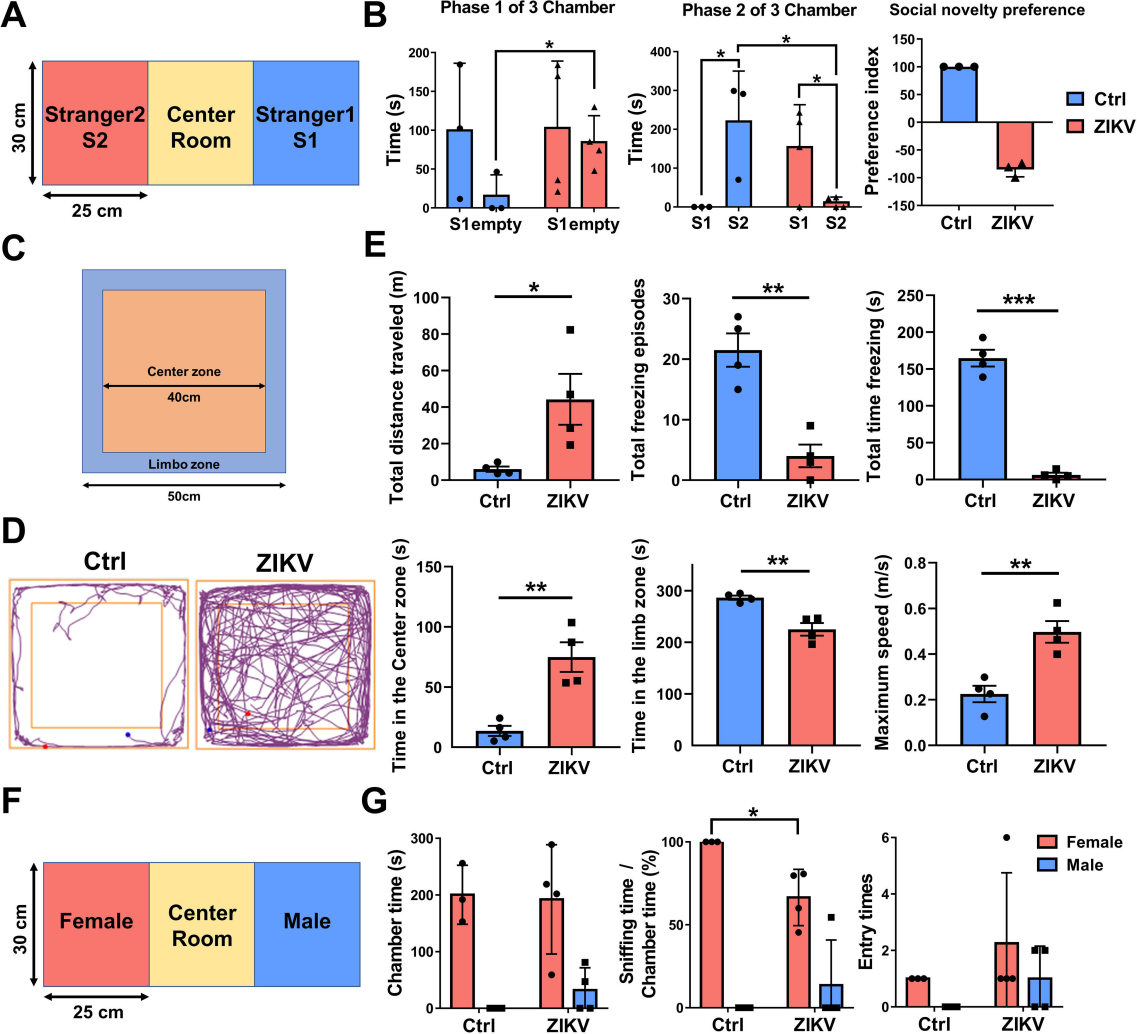
Neonatal ZIKV infection caused abnormal behaviors in mice. (**A**) Schematic diagram of the three-chamber task to evaluate social behavior and social novelty preference of mice. (**B**) The time spent on socializing with Stranger 1 or Stranger 2 and index of social novelty preference for ZIKV-infected and control mice (*n* = 3 to 4) at 12 weeks during the three-chamber task (*P < 0.05; Student’s *t*-test). (**C**) Schematic diagram used in the open field test to evaluate the motor ability and autonomous exploration. (**D**) Representative moving tracks of ZIKV-infected and control mice at 12 weeks. (**E**) Statistics of total distance traveled, total freezing episodes, total time freezing, time in the center zone, time in the limb zone, and the maximum speed of ZIKV-infected and control mice (*n* = 4) during the open field test (*P < 0.05; **P < 0.01; ***P < 0.001; Student’s *t*-test). (**F**) Schematic diagram of the three-chamber task to evaluate the sex preference of male mice. (**G**) Statistics of the chamber time, sniffing time, and entry times of ZIKV-infected and control mice (*n* = 3 to 4) at 12 weeks during the three-chamber task (*P < 0.05; Student’s *t*-test).

To determine whether ZIKV infection affects social behavior in mice, we used the three-chamber task to inspect the voluntary initiation of social interaction and preference for social novelty of mice (Fig. 5A). In the sociability phase, one partner (Stranger 1) was placed in a chamber on one side, and the chamber on the other side was empty. Control mice tended to spend more time in the chamber with social partner and barely entered the empty chamber. By contrast, ZIKV-infected mice spent significantly more time in the empty chamber (Fig. 5B). In the social novelty phase, a novel partner (Stranger 2) was introduced into the previously empty chamber. Control mice exhibited a preference for the novel partner (Stranger 2), while ZIKV-infected mice displayed a preference for the familiar partner (Stranger 1) (Fig. 5B). ZIKV-infected mice had a remarkably lower index of social novelty preference (Fig. 5B). These results suggested that ZIKV-infected mice had reduced sociability and preference for social novelty.

Next, we used the open field test to evaluate the motor ability and autonomous exploration ability of mice (Fig. 5C). Each test mouse was placed in a 50 × 50 cm square cage to move freely. Compared with control mice, ZIKV-infected mice had longer distance traveled, fewer freezing episodes, less freezing time, and higher maximum speed (Fig. 5D and E). ZIKV-infected mice spent more time traveling in the central area than control mice and spent less time traveling in the peripheral area (Fig. 5D and E). These results indicated reduced autonomous exploration behavior and increased aimless running in ZIKV-infected mice, while their motor ability was normal.

Finally, we used the three-chamber task to evaluate whether ZIKV infection altered the sexual preference of male mice leading to prolonged time to delivery. One male partner and one female partner were, respectively, introduced into the left and right chambers (Fig. 5F). Control male mice consistently entered the female chamber and kept sniffing with the female partner at all times (Fig. 5G). By contrast, ZIKV-infected male mice shuttled repeatedly through three chambers, and they spent significantly less time sniffing with the female partner (Fig. 5G). These results indicated that ZIKV-infected male mice exhibited a decreased preference for female mice, which might cause reduced mating behavior and thus prolonged time to delivery.

### ZIKV infection in the hypothalamus causes long-term hormone deficiencies in the HPG axis

**Fig 6:**
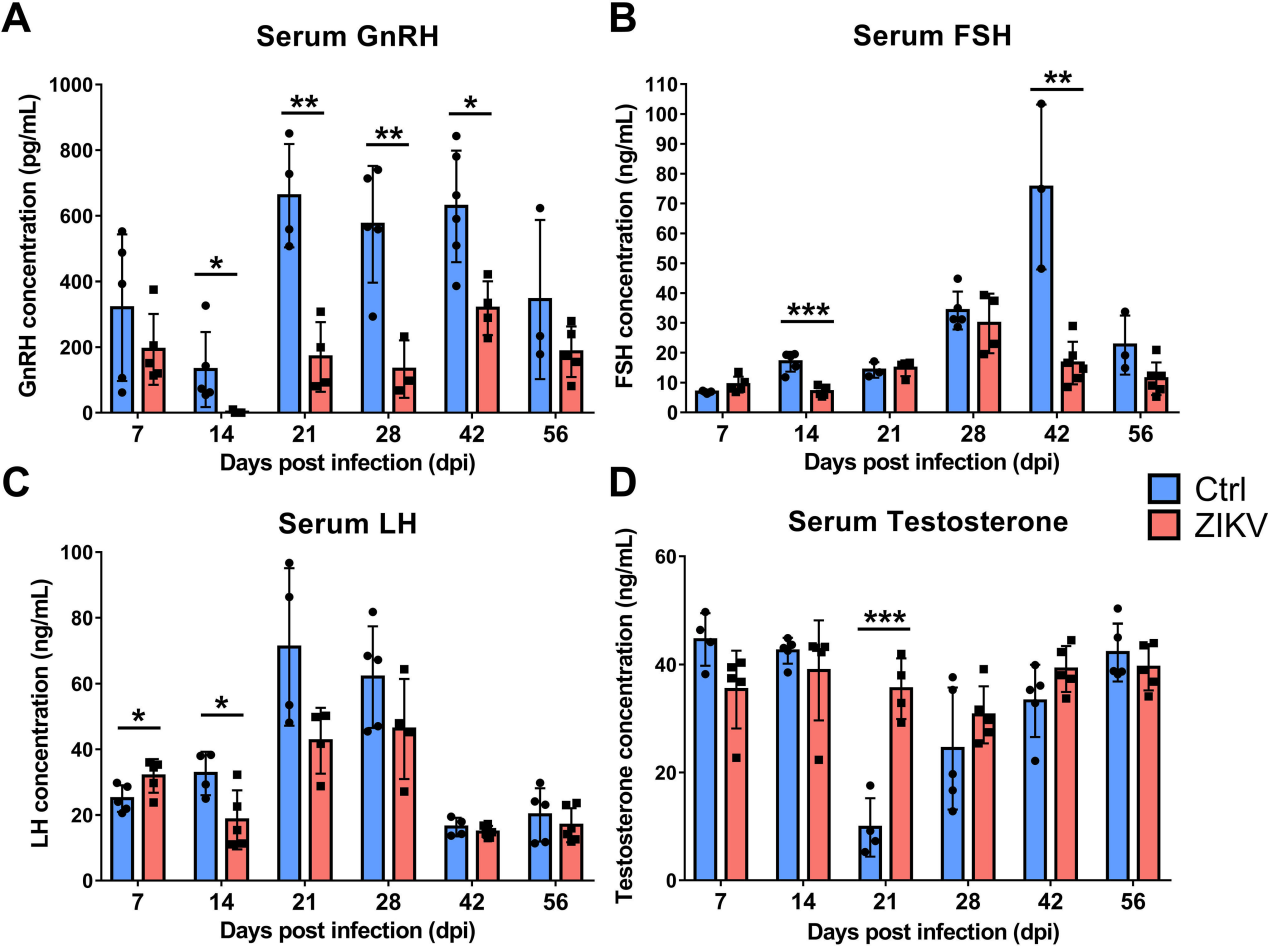
Serum GnRH, FSH, LH, and testosterone concentrations of ZIKV-infected and control mice (*n* = 3 to 6 for each time point) determined by ELISA (*P < 0.05; **P < 0.01; ***P < 0.001; Student’s *t*-test).

The hypothalamus regulates hormone production via the HPG axis, affecting reproductive development and mating behavior. To determine whether ZIKV infection affects hormone secretion of the HPG axis, we used enzyme-linked immunosorbent assay (ELISA) to assay serum concentration of four hormones, including gonadotropin-releasing hormone (GnRH), follicle-stimulating hormone (FSH), luteinizing hormone (LH), and testosterone. Compared with control mice, ZIKV-infected mice had significantly lower GnRH concentrations from 7 to 56 dpi (Fig. 6A), and lower FSH and LH concentrations from 14 to 56 dpi (Fig. 6B and C). Control mice had two peaks of testosterone concentrations at 7 and 56 dpi, which is consistent with physiological testosterone surges as reported (25). By contrast, ZIKV-infected mice displayed sustained high levels of testosterone concentration, which was slightly below the testosterone peaks of control mice (Fig. 6D). These results indicated long-term hormone deficiencies of the HPG axis in ZIKV-infected mice.

**Fig 7:**
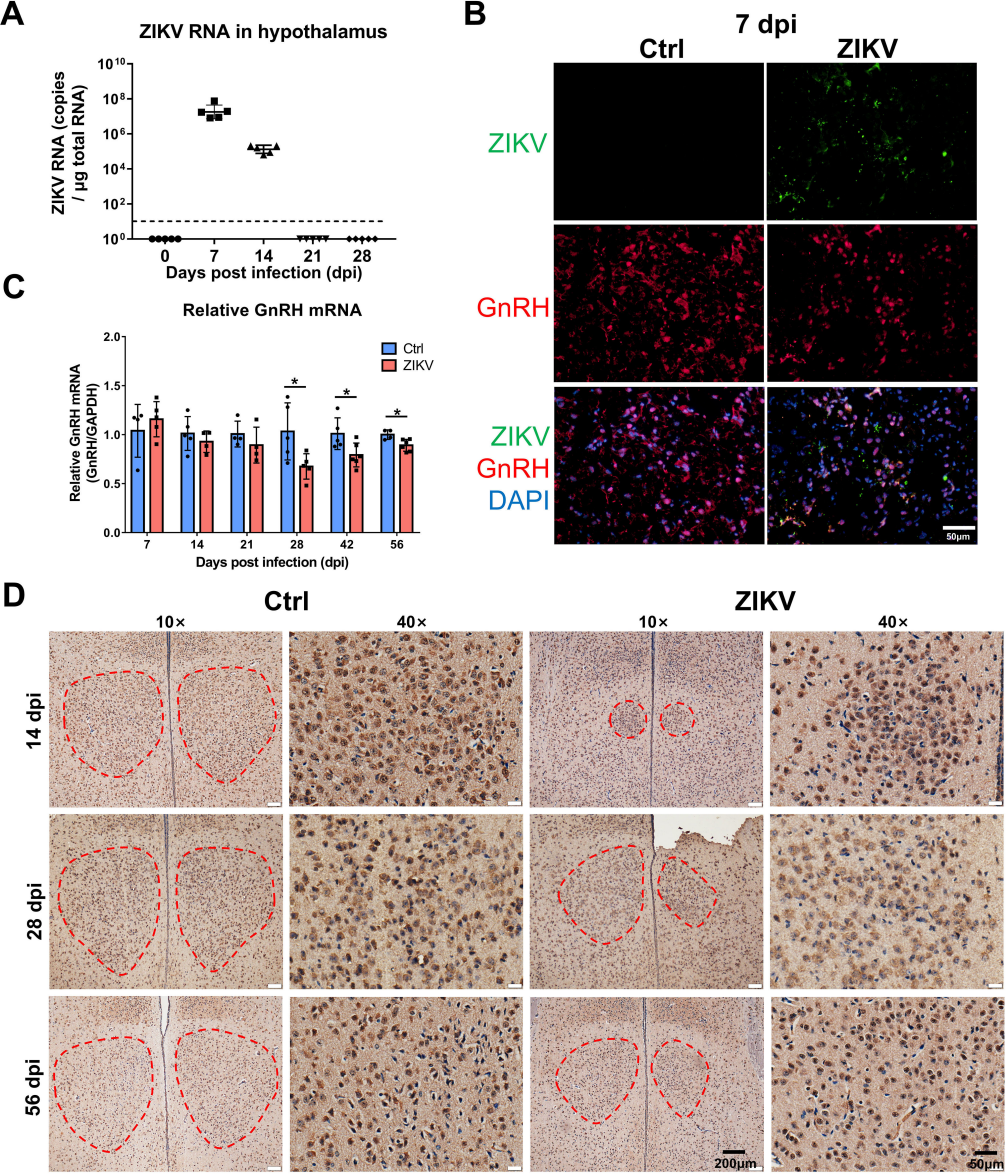
ZIKV infection in the hypothalamus caused decreased GnRH expression in mice. (**A**) Hypothalamus collected from ZIKV-infected mice (*n* = 5 for each time point) were analyzed for viral RNA by RT-qPCR. The dashed line indicates the detection limit. (**B**) Immunofluorescence staining for ZIKV and GnRH in the hypothalamus of ZIKV-infected and control mice at 7 dpi. (**C**) The relative GnRH mRNA expression in the hypothalamus of ZIKV-infected and control mice (*n* = 4 to 6 for each time point) was assayed by RT-qPCR (*P < 0.05; Student’s *t*-test). (**D**) Immunohistochemical staining for GnRH in the hypothalamus of ZIKV-infected and control mice at 10× (left) and 40× (right) magnification. Red dashed circles indicate the area of GnRH^+^ neuroendocrine cells.

**Fig 8:**
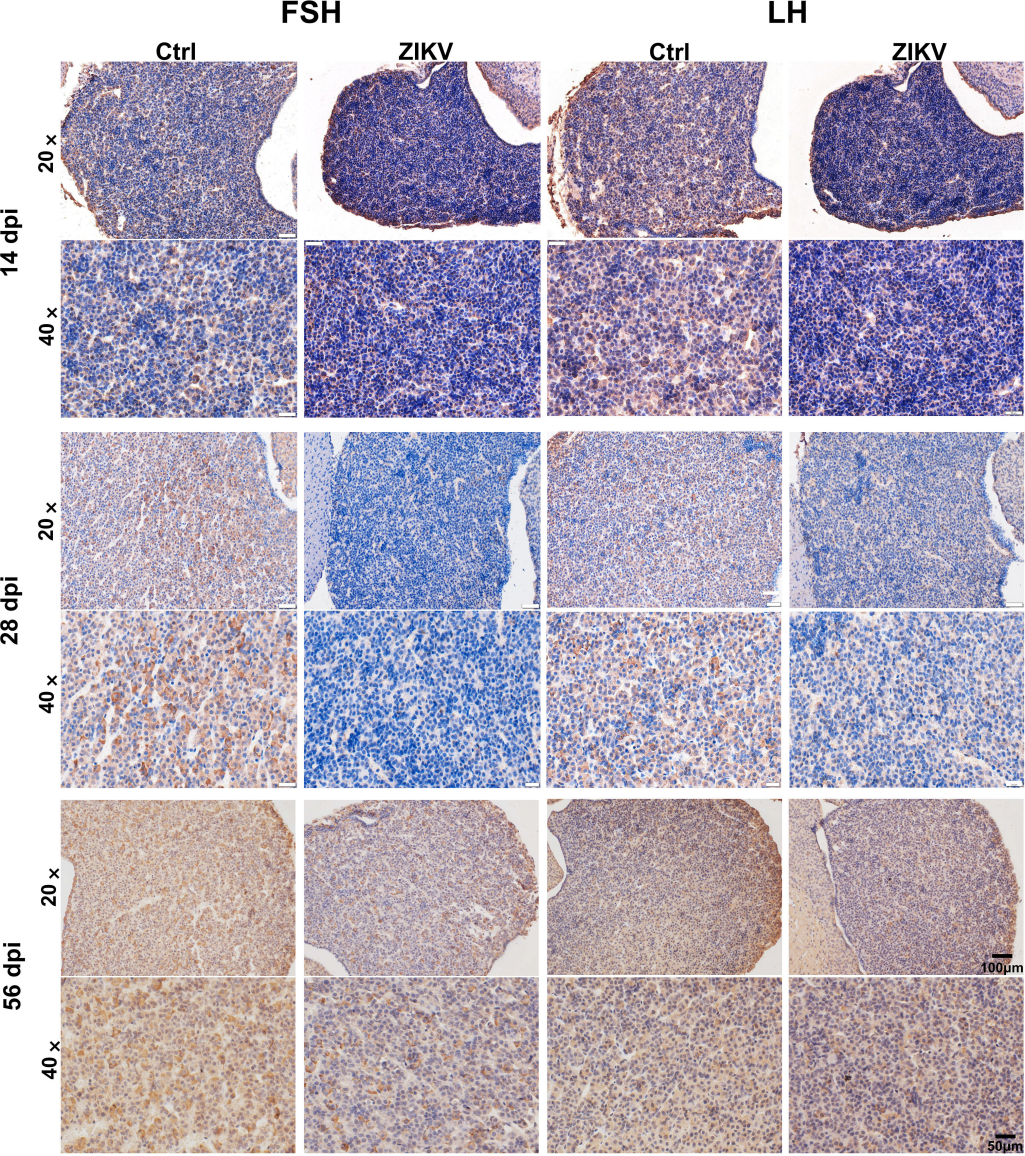
Immunohistochemical staining for FSH and LH in the pituitary of ZIKV-infected and control mice at 14, 28, and 56 dpi.

To determine the mechanisms underlying hormone deficiencies of the HPG axis, we examined the hypothalamus and pituitary in mice. ZIKV RNA was detected in the hypothalamus from 7 to 14 dpi (Fig. 7A), while it was undetectable in the pituitary at any time point. Colocalization of ZIKV and GnRH^+^ cells was also observed in the hypothalamus of mice at 7 dpi (Fig. 7B). ZIKV-infected mice displayed reduced GnRH RNA in the hypothalamus than control mice from 14 to 56 dpi (Fig. 7C). Immunohistochemical staining also revealed a reduced area of GnRH^+^ cell mass in the hypothalamus of ZIKV-infected mice (Fig. 7D). ZIKV-infected mice showed lighter staining for FSH and LH in the pituitary (Fig. 8). These results indicated that ZIKV could infect the hypothalamus of mice, causing delays in neuroendocrine development and decreased hormone expressions of the HPG axis.

**Fig 9:**
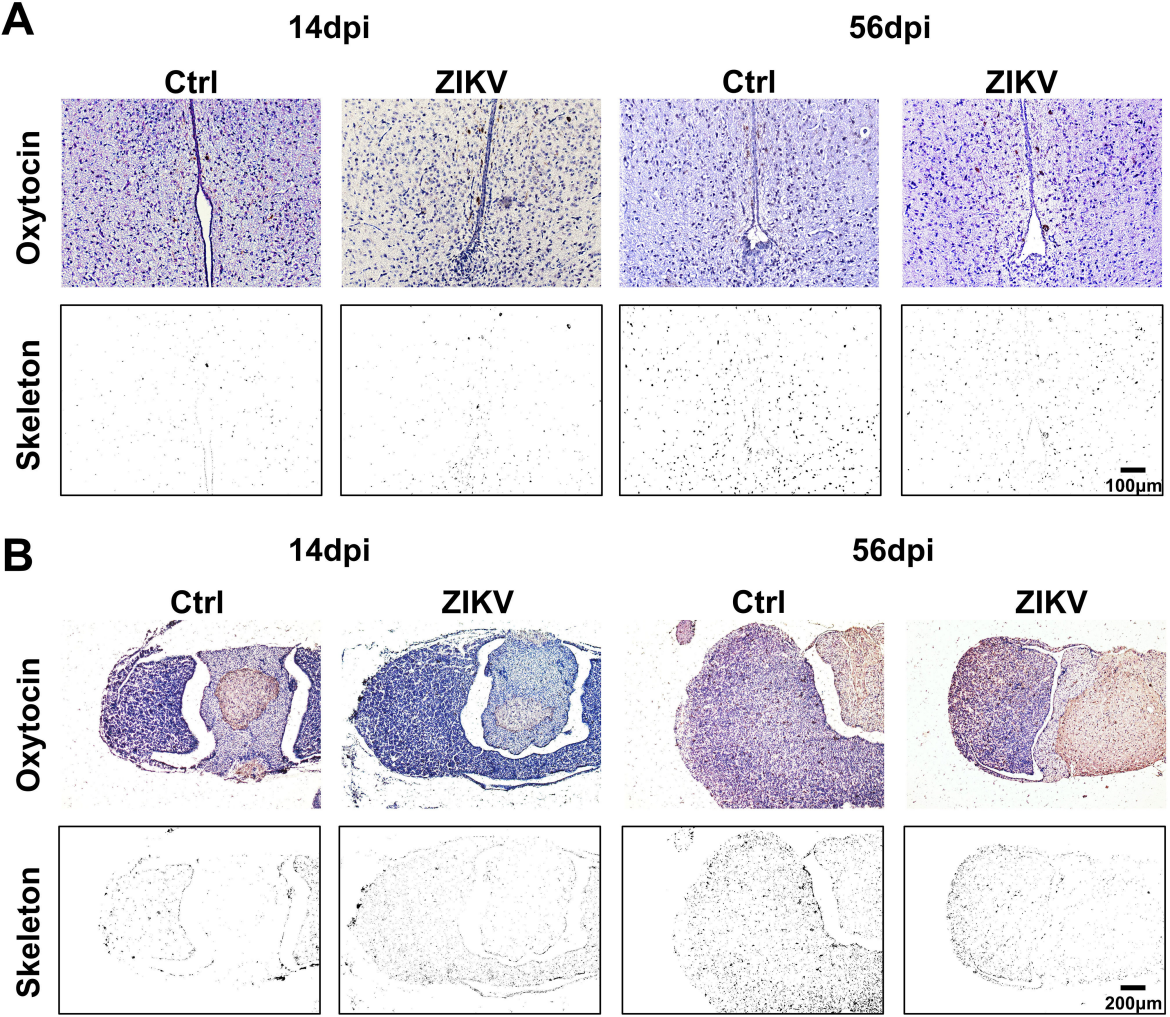
Effects of ZIKV infection on oxytocin production. (**A**) Immunohistochemical staining for oxytocin in the hypothalamus of ZIKV-infected and control mice at 14 and 56 dpi. Scale bar = 100 µm. (**B**) Immunohistochemical staining for oxytocin in the pituitary of ZIKV-infected and control mice at 14 and 56 dpi. Scale bar = 200 µm. Skeleton indicates positive staining for oxytocin extracted by skeleton analysis of ImageJ software.

**Fig 10:**
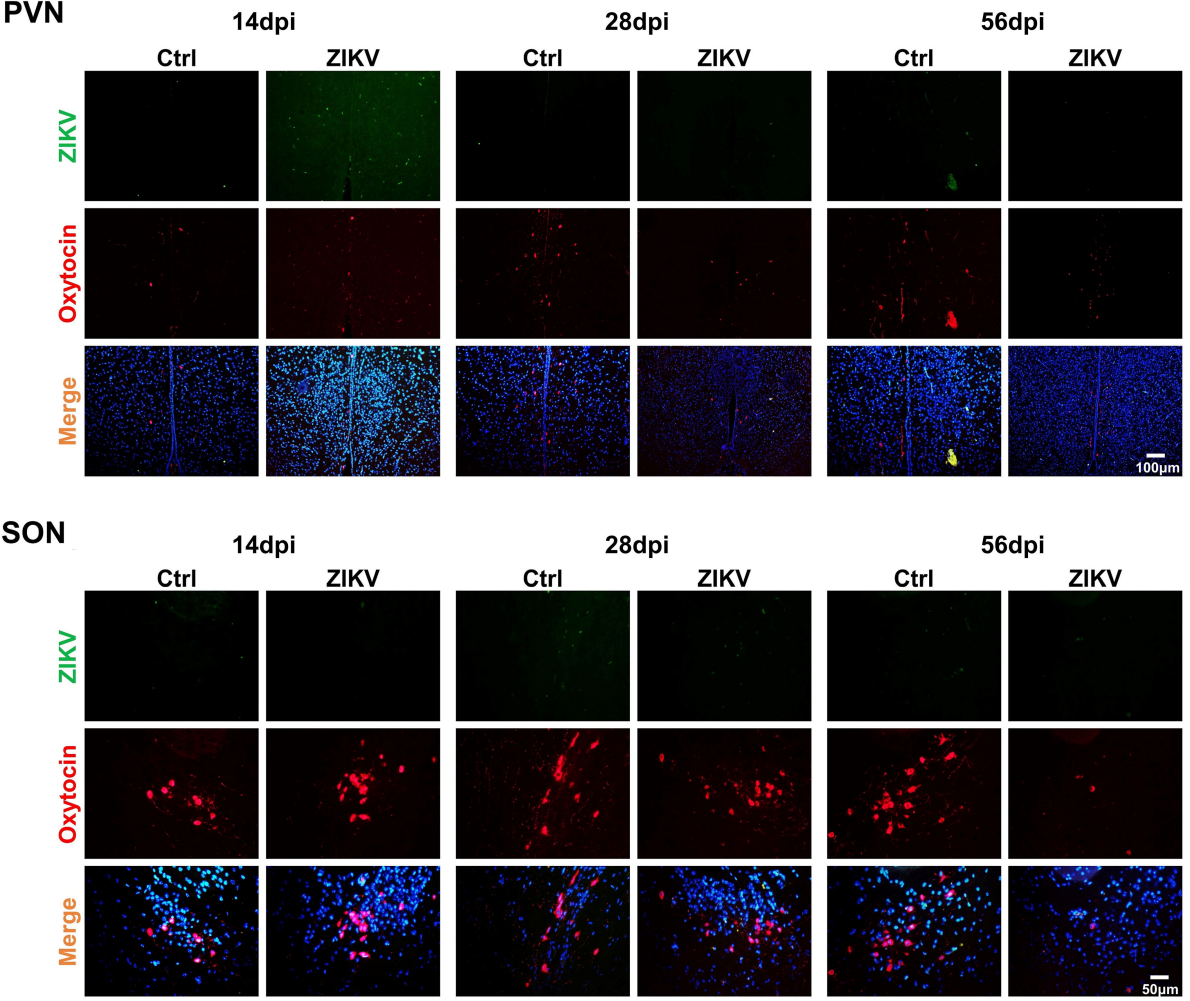
Immunofluorescent staining for ZIKV and oxytocin in the paraventricular nucleus (PVN) and supraoptic nucleus (SON) of ZIKV-infected and control mice at 14, 28, and 56 dpi.

Oxytocin secreted by the hypothalamus can regulate social behavior (26, 27). To determine whether ZIKV infection affects oxytocin production, we used immunostaining for oxytocin. The hypothalamus and pituitary of ZIKV-infected mice displayed significantly reduced OXT^+^ cells at 56 dpi (Fig. 9A and B). The reduced OXT^+^ cells were also observed in the supraoptic nucleus and paraventricular nucleus of ZIKV-infected mice at 28 and 56 dpi (Fig. 10). No co-staining of ZIKV and OXT was observed (Fig. 10). These results suggested that ZIKV infection reduced oxytocin expressions, which might be associated with abnormal social behavior in mice.

### Transcriptome characteristics of hypothalamus and testes in ZIKV-infected mice

**Fig 11:**
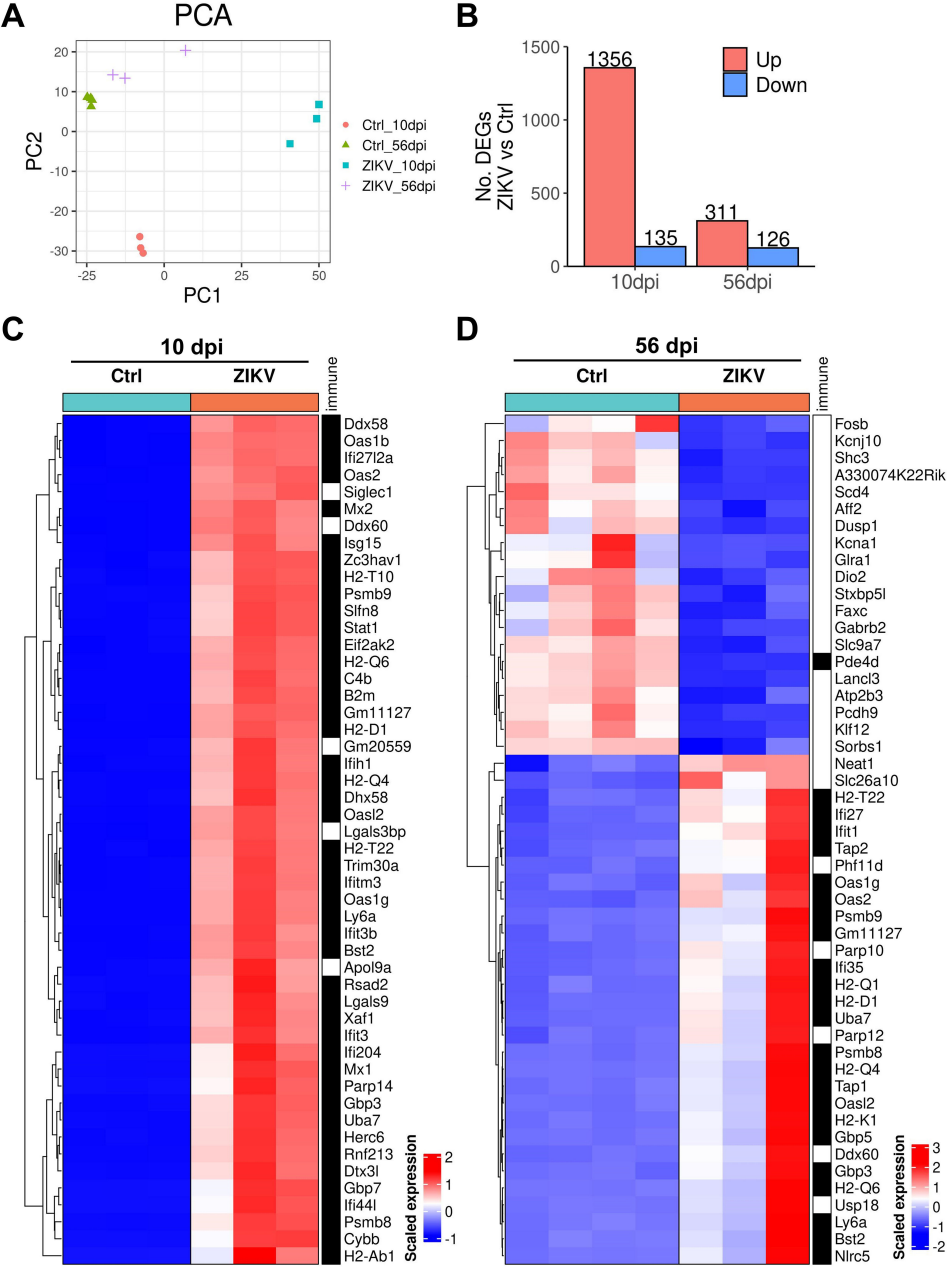
Transcriptome characteristics of the hypothalamus from neonatal ZIKV-infected mice. Hypothalamic samples collected from ZIKV-infected and control mice at 10 and 56 dpi were subjected to RNA-seq. (**A**) PCA plots of hypothalamic samples were collected from ZIKV-infected and control mice at 10 and 56 dpi. (**B**) The number of up-regulated and down-regulated DEGs (ZIKV group vs Ctrl group) at 10 and 56 dpi (FDR-adjusted *P*-value < 0.05, |log2 fold change| > 1). (**C and D**) Heatmaps of differentially expressed genes (Top 50) from the transcriptome of the hypothalamus in ZIKV-infected and control mice at 10 dpi (**C**) and 56 dpi (**D**) (FDR-adjusted *P*-value < 0.05, |log2 fold change| > 1). The black blocks in the right annotation indicate genes associated with immune response.

**Fig 12:**
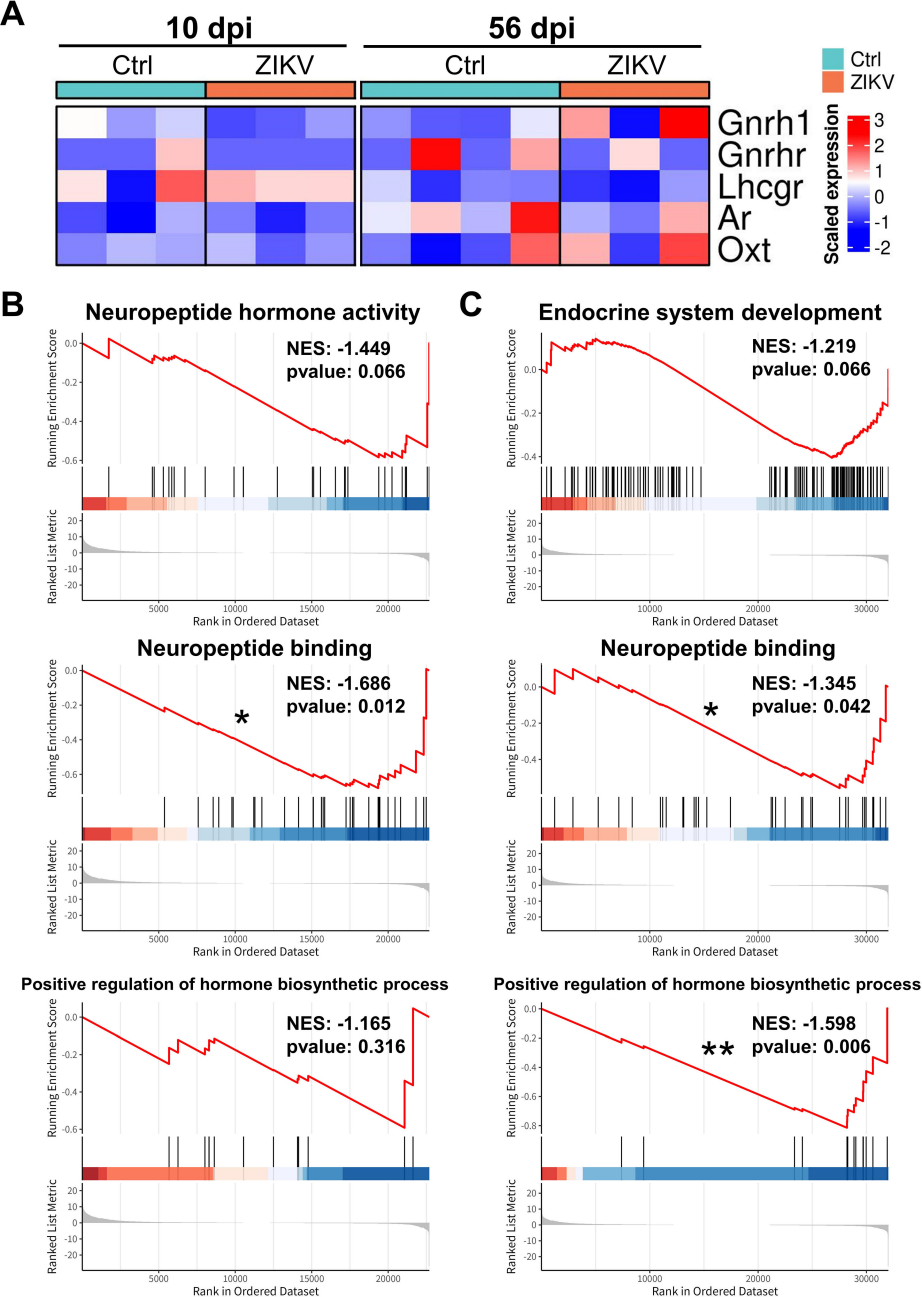
Transcriptome analysis of neuroendocrine genes in the hypothalamus of ZIKV-infected mice. (**A**) Heatmaps of genes related to the HPG axis from the transcriptome of the hypothalamus in ZIKV-infected and control mice at 10 and 56 dpi. (**B and C**) GSEA diagrams associated with the neuroendocrine system in the transcriptome of the hypothalamus at 10 dpi (**B**) and 56 dpi (**C**) (*P < 0.05; **P < 0.01; permutation test).

To further investigate the effects of ZIKV infection on the hypothalamus, we performed RNA-seq to analyze transcriptome changes in the hypothalamus of mice. Hypothalamus samples were collected from ZIKV-infected and control mice at 10 and 56 dpi. The difference in hypothalamic transcriptome between ZIKV-infected and control mice was remarkable at 10 dpi, and decreased at 56 dpi, albeit still significant (Fig. 11A). We found more differentially expressed genes (DEGs) at 10 dpi (including 1356 up-regulated DEGs and 135 down-regulated DEGs) than at 56 dpi (including 311 up-regulated DEGs and 126 down-regulated DEGs), most of which are related to immune response (Fig. 11B through D). Decreased GnRH and oxytocin expression levels were also observed in ZIKV-infected mice at 10 dpi (Fig. 12A), but there were no significant differences in the expression levels of hormone-related genes (Fig. 12A), which may be due to their low expression levels being overshadowed by differences in immune-related genes. Based on gene set enrichment analysis (GSEA), we found that “Neuropeptide hormone activation,” “Endocrine system development,” “Neuropeptide binding,” and “Positive regulation of hormone biosynthetic process” were inhibited in ZIKV-infected mice (Fig. 12B and C), which may explain the reduced production and secretion of GnRH and oxytocin.

**Fig 13:**
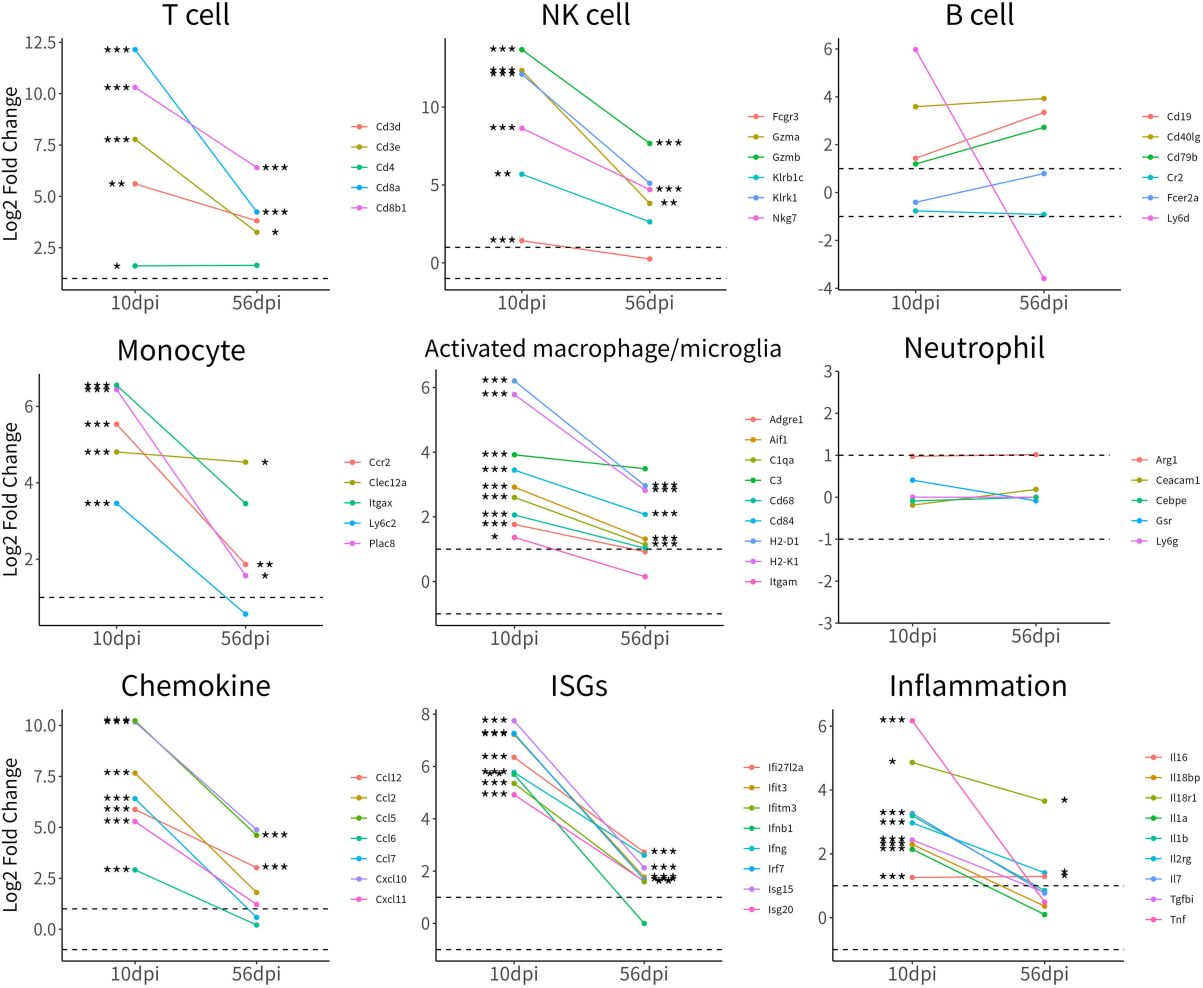
Line graph of T-cell biomarkers, NK cell biomarkers, B-cell biomarkers, monocyte biomarkers, activated macrophage/microglia biomarkers, neutrophil biomarkers, chemokine genes, interferon-stimulated genes (ISGs), and inflammatory genes. Shown are the change of log2 fold change values relative to control mice at 10 and 56 dpi. The dashed lines represent up-regulated and down-regulated gene thresholds (|Log2FC| > 1). The asterisk (*) represents the statistical difference between the ZIKV-infected group and the control group (*P < 0.05; **P < 0.01; ***P < 0.001; Wald test).

Previous studies have shown that the development of the neuroendocrine system is negatively affected by inflammation (28 – 30), so we monitor dynamic changes in expression levels of genes related to the inflammatory response (Fig. 13). We observed significantly increased gene expression levels in T cells (e.g., Cd3e, Cd8a, Cd4), NK cells (e.g., Nkg7, Fcgr3, Gzma), and monocytes (e.g., Ccr2, Itgax, Ly6c2) but not B cells and neutrophils in the ZIKV-infected hypothalamus at 10 dpi. The expression levels of genes associated with macrophage and microglia activation were also significantly increased, including Cd68, Cd84, C1qa, C3, Aif1, and H2-D1. In addition, the expression levels of chemokines (e.g., Ccl2, Ccl5, and Ccl12), interferon-stimulated genes (e.g., Isg15, Ifi27l2a, and Irf7), and inflammatory factors (e.g., Ifng, Il1b, Tnf, and Tgfbi) also increased significantly at 10 dpi. Surprisingly, although differences in immune-related genes decreased over time, infiltration of CD8^+^ T cells, NK cells, and monocytes, activation of macrophages/microglia, and inflammatory responses persisted up to 56 dpi (Fig. 13), which may have had a long-term negative effect on the development of hypothalamic neuroendocrine cells.

**Fig 14:**
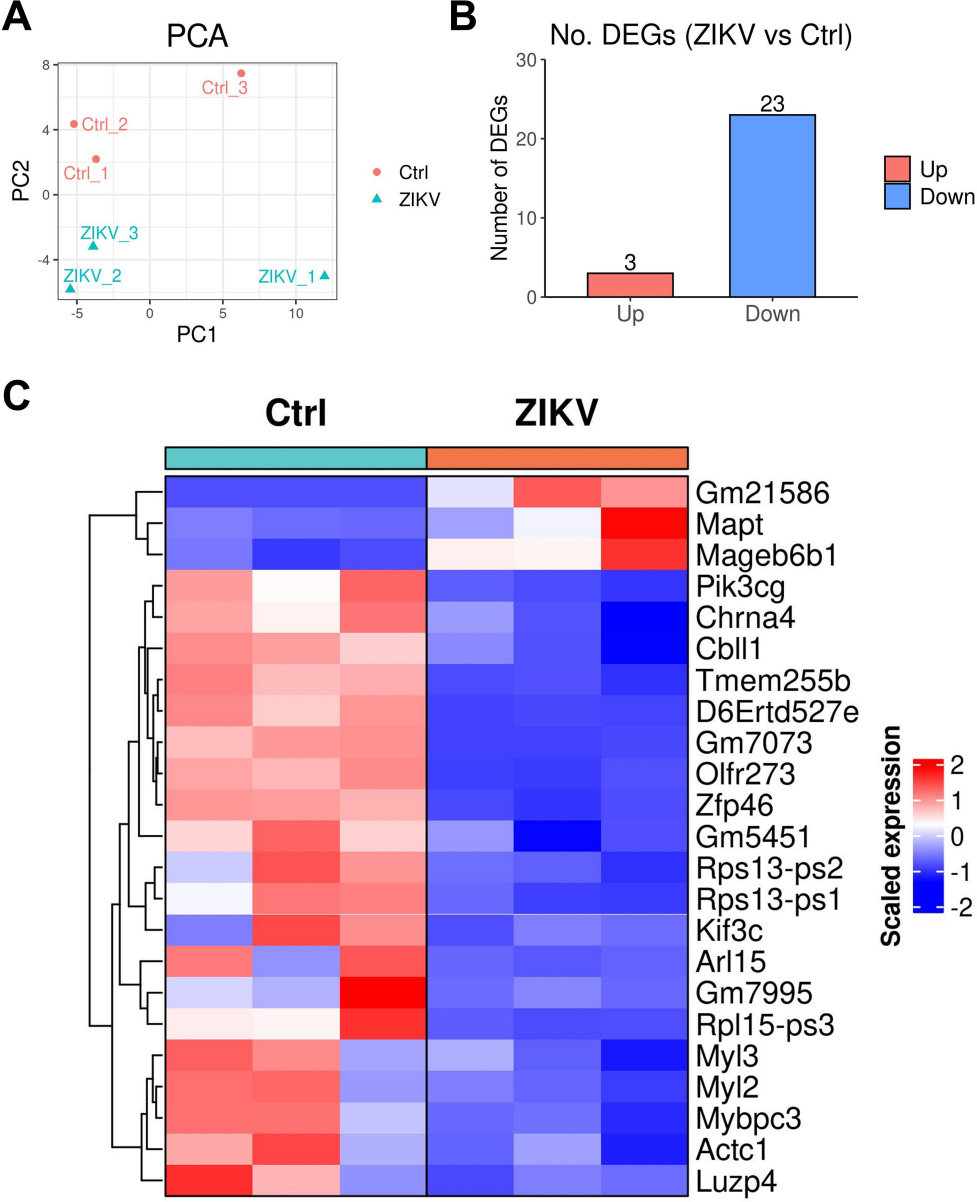
Transcriptome characteristics of testis from neonatal ZIKV-infected mice. Testis samples collected from ZIKV-infected and control mice at 56 dpi were performed by RNA-seq. (**A**) PCA plots of testis samples collected from ZIKV-infected and control mice at 56 dpi. (**B**) The number of up-regulated and down-regulated DEGs (ZIKV group vs Ctrl group) at 56 dpi (FDR-adjusted *P*-value < 0.05, |log2 fold change| > 1). (**C**) Heatmaps of all differentially expressed genes from the transcriptome of testis in ZIKV-infected and control mice.

Finally, we performed RNA-seq on testes at 56 dpi to follow the long-term consequences of testes from ZIKV-infected mice. ZIKV-infected and control mice had similar testicular transcriptome levels at 56 dpi (Fig. 14A). There were three up-regulated DEGs and 23 down-regulated DEGs in the testes of ZIKV-infected mice (Fig. 14B). Most down-regulated DEGs were related to metabolism, including Zfp46, Arl15, Rps13-ps1, and Rps13-ps2 (Fig. 14C). Lower Cbll1 (encodes the E3 ubiquitin ligase of the E-cadherin complex) expression might reduce the strength of cell adhesion. Higher Mapt expression could promote microtubule assembly and stability (31). In addition, ZIKV-infected testes showed no abnormal immune and reproductive genes. Generally, ZIKV-infected mice had nearly normal transcriptome in the testis at 56 dpi, which is distinct from significant transcriptome changes in the hypothalamus.

Taken together, ZIKV could damage the hypothalamus of mice for a longer time, and immune responses persisted even up to 56 dpi. By contrast, ZIKV just transiently affected the testes of ZIKV-infected mice and did not disturb expressions of its spermatogenic genes.

## DISCUSSION

Newborns exposed to ZIKV have received global attention because of their developmental complications, including delays in cognitive, language, and motor development (12 – 15) as well as cryptorchidism (16, 17). However, the impacts of ZIKV infection on the reproductive health of these children remain unclear. In the current study, using a mouse model, we demonstrated that long-term hormone deficiencies of the HPG axis caused by postnatal ZIKV infection resulted in not only decreased sperm quality and quantity but also abnormal behavior, ultimately leading to diminished fertility.

In this study, male BALB/c suckling mice were inoculated with ZIKV at postnatal day 2, which is equivalent to the neonatal infection during the third trimester (19, 20). This animal model was widely used to study developmental delays of newborns exposed to ZIKV (18, 20). For example, Zhao et al. (20) found that postnatal ZIKV-infected mice had neurological deficits in social interaction, learning, and memory, which was consistent with neurodevelopmental deficits observed in children perinatally exposed to ZIKV in Brazil (12). In comparison to immunodeficient mice (e.g., *Ifnar1^-/-^* mouse), this neonatal mouse model better mimics human diseases, suggesting that it may be more suitable for studying congenital ZIKV syndrome.

Using the neonatal mouse model, we observed brain atrophy, abnormal social behaviors, and stereotypic behaviors in ZIKV-infected mice, which were consistent with the observations in several ZIKV-infected animal models reported previously (20, 32, 33). Previous studies have focused primarily on the cortex and the visual system and found that all kinds of central nervous system (CNS) cells were susceptible to ZIKV infection, exhibiting cell death and decreased neurogenesis (34 – 36), which were thought to be a major contributor of various neurodevelopmental complications in ZIKV infection. However, the effects of ZIKV infection on the hypothalamus have hardly been studied. In this study, we detected the presence of ZIKV in the hypothalamus, which was consistent with our previous study (18, 37). As the center of the neuroendocrine system, the hypothalamus can release hormones to regulate various important physiological processes, such as GnRH regulating reproductive development via the HPG axis (38) and oxytocin regulating social behaviors (26, 27). Both GnRH and oxytocin secreted by the hypothalamus were significantly reduced in ZIKV-infected mice, which was highly correlated with persistent damage of ZIKV infection. We speculated that the long-term decreased hormones secreted by the hypothalamus contribute to the delayed testicular development, abnormal sperm, abnormal social behaviors, and decreased fertility, which for the first time linked the hypothalamus to the reproductive system and social behaviors after ZIKV infection.

GnRH can directly act on anterior pituitary cells to regulate releases of FSH and LH (39, 40). In this study, serum FSH and LH concentrations had a long-term decrease, albeit transiently increased within the first week after infection, which was possibly attributed to the break of negative feedback balance of the HPG axis caused by persistent GnRH reduction. FSH can promote the proliferation and development of spermatogenic cells and Sertoli cells (41, 42). In this study, the lower serum FSH concentration was possibly the reason for reversible developmental delays of spermatogenic cells and Sertoli cells in the testes of ZIKV-infected mice. In addition, spermatogenesis depends primarily on the stimulation of FSH, LH, and testosterone (41, 43, 44). FSH can promote spermatogenesis directly by stimulating spermatogenic cells (41, 43, 44). LH deficiency could result in azoospermia, as revealed by Abel et al. (45). Consequently, persistent lower FSH and LH levels in the study most likely led to decreases in sperm quantity and quality of mice, which resulted in decreased fertility and decreased offspring quality.

In previous studies, ZIKV infection can cause severe damage to testicular morphology and massive inflammatory cell infiltration in C57BL/6 mice treated with anti-Ifnar1 blocking monoclonal antibody (24) or Ifnar1^-/-^ mice (22, 23), and ZIKV infection was thought a major contributor of the testicular damage. In the current study, ZIKV was detected in only 40% (2/5) of BALB/c mouse testes, and there was no serious damage and inflammatory cell infiltration, which was distinct from the immunodeficient mice mentioned above. Considering the low infection rate of testes but sperm abnormalities when ZIKV was eliminated, we speculated that testicular developmental delays and oligospermia observed in BALB/c mice were primarily attributed to hormone deficiencies of the HPG axis rather than the direct attack of ZIKV to testes or the immune injury.

The diminished fertility may be associated with many factors in mice. On the one hand, hormone disorders of the HPG axis led to decreases in sperm quantity and quality in ZIKV-infected mice, which directly affects the fertility rate and quality of offspring mice. On the other hand, the decreased preference for females and stereotyped behaviors observed in ZIKV-infected mice reduced their mating behavior, which might be another important cause for the prolonged time to delivery. Therefore, in future experiments, administering GnRH or oxytocin may be a way to improve fertility by regulating hormone levels of the HPG axis or mating behavior.

Healthy male mammals usually experience several physiological testosterone elevations: the first at embryonic day 13 of mice or the 7th week of pregnancy in humans, the second in neonates, and the third at puberty (25). After a neonatal testosterone surge, testosterone drops to a low level until puberty (25). In this study, however, ZIKV-infected mice did not show a physiological decrease in serum testosterone from birth till adulthood, the pathogenesis of which remains unclear and may be related to the blocked consumption of testosterone. As a protective hormone against ZIKV infection (46), whether disordered testosterone level in postnatal ZIKV-infected mice affects disease progression needs to be further studied.

In summary, we comprehensively clarified the effects of postnatal ZIKV infection on fertility from multiple perspectives including the HPG axis, testis, sperm, and abnormal behaviors, revealing that postnatal ZIKV infection caused long-term hormone deficiencies and diminished fertility in mice. Although the extent to which these observations in mice translate to humans remains unclear, these findings did suggest that the reproductive health and hormone levels of ZIKV-exposed children should receive more attention to improve their living quality.

## MATERIALS AND METHODS

### Cells and virus

C6/36 cells were cultured in RPMI 1640 medium (Gibco, USA) with 10% fetal bovine serum (FBS, PAN, Germany) and maintained at 28°C. Vero cells were cultured in minimum essential medium (MEM, Gibco, USA) with 5% FBS and maintained at 37°C. ZIKV (strain SMGC-1, Asian lineage, GenBank accession number: KX266255, ) was propagated in C6/36 cells and titrated on Vero cells under overlay medium containing 1.2% methylcellulose by plaque assay. Stocks were stored at 80°C until use.

### Mouse

Pregnant BALB/c mice from the Beijing Vital River Laboratory Animal Technology Co., Ltd. (Beijing, China) were bred and maintained under a specific pathogen-free animal facility at Capital Medical University.

### Mouse experiments

Mouse pups at postnatal day 2 were inoculated from the brain λ point with 100 plaque-forming units of ZIKV or an equal volume of phosphate-buffered saline (PBS). The body weight and survival rate of mice were recorded for 56 days. Hypothalamus, pituitary, and testis were collected at 7, 10, 14, 21, 28, 42, and 56 dpi to study morphology, ZIKV RNA, and transcriptome. To determine the effects of ZIKV infection on fertility, ZIKV-infected and control male mice were mated with healthy age-matched female mice in a ratio of 1:2 when they were 6 or 8 weeks old. The 6-week mating experiment and the 8-week mating experiment were independent experiments in different batches. The pregnancy rate and time from being co-caged to delivery were observed for 50 days. The body weight and sex ratio of offspring mice were recorded at postnatal days 0 and 28.

### ZIKV RNA quantification and GnRH mRNA relative quantification

Hypothalamus, pituitary, and testis were harvested from ZIKV-infected and control mice at different time points as indicated. RNA was extracted from tissue lysates by Trizol (Transgen China, ET101-01) according to the manufacturer’s protocol. Real-time quantitative PCR (RT-qPCR) was performed as previously reported (18) by Quant One Step RT-qPCR (Tiangen, China) on a 7500 Real-Time PCR System (Applied Biosystems, USA). ZIKV RNA copies of samples were quantified as the copy number per microgram of total RNA by the standard curve method. ZIKV RNA copies transcribed *in vitro* were quantified and used as a standard template to establish the standard curve. Relative quantification of GnRH mRNA was determined by the 2^−ΔΔCt^ method. The primer sequences were as follows: ZIKV forward: 5′-TCAGACTGCGACAGTTCGAGT-3′; ZIKV reverse: 5′-GCATATTGACAATCCGGAAT-3′; GAPDH forward: 5′-GCATTGTGGAAGGGCTCA-3′; GAPDH reverse: 5′-ACCAGTGGATGCAGGGAT-3′; GnRH forward: 5′-AGCACTGGTCCTATGGGTTG-3′; GnRH reverse: 5′-GGGGTTCTGCCATTTGATCCA-3′ (47).

### ELISA

The serum GnRH, FSH, LH, and testosterone concentrations in mice were quantified by ELISA, according to the manufacturer’s instructions. ELISA reagents of GnRH, FSH, and LH were purchased from Cloud-Clone (mouse GnRH, FSH, and LH ELISA Kits, Cloud-Clone, China). Briefly, 100 µL of standard dilution or serum dilution was added into each well, then the plate was incubated for 1 h at 37°C. Subsequently, 50 µL of Detection Reagent A was added and the plate was incubated for 1 h at 37°C. After washing, 50 µL of Detection Reagent B was added and the plate was incubated for 30 min. Finally, 90 µL of Substrate Solution was added and the plate was incubated at 37°C for 15 min, followed by the addition of 50 µL of Stop Solution. The absorbance was measured at 450 nm in a Multiskan spectrum 1500 (Thermo, USA). ELISA reagents of testosterone were purchased from Enzo Life Sciences (Testosterone ELISA kit, Enzo Life Sciences, USA). Briefly, 100 µL of standard dilution or serum dilution and 50 µL of yellow Antibody were added into each well, then the plate was incubated for 1 h at 500 rpm. Subsequently, 50 µL of blue Conjugate was added and the plate was incubated for 1 h at 500 rpm. After washing, 200 µL of the pNpp Substrate solution was added, and the plate was incubated for 1 h at 37°C. Finally, 50 µL of Stop Solution was added. The absorbance was measured at 405 nm in a Multiskan spectrum 1500 (Thermo, USA).

### Immunohistochemistry staining

To assay hormone expressions in the hypothalamus and pituitary, tissues were fixed in 4% paraformaldehyde solution overnight, then dehydrated, and paraffin-embedded. Tissue sections were incubated with rabbit polyclonal anti-GnRH (PAA843Mu01, Cloud-Clone, China), anti-FSH (PAA830Mu01, Cloud-Clone, China), anti-LH (PAA441Ra01, Cloud-Clone, China), and anti-Oxytocin (ab212193, Abcam) overnight. After washing, the slides were stained with a secondary HRP-conjugated goat anti-rabbit antibody (Zhongshan Golden Bridge Bio Co., Ltd., China) for 1 h. The reaction was visualized by the addition of 3,30-diaminobenzidine (DAB) as a chromogen and then stopped by removing the DAB and then it was washed with running water. Images were captured with the Olympus microscope (IX71, Olympus, Japan). Skeleton analysis was performed using ImageJ.

### Hematoxylin and eosin staining

To investigate pathological changes in ZIKV-infected testes, testicular sections were subjected to HE staining. Testicular sections were immersed into xylene and alcohol, then stained with hematoxylin for 12 min. After being stained with eosin for 20 min and re-immersed in alcohol and xylene, sections were mounted by synthetic resin.

### Immunofluorescence staining

Tissue sections were incubated with mouse anti-ZIKV NS1 monoclonal antibody (1:500, GT5212, Invitrogen), mouse anti-DDX4 antibody (1:400, ab27591, Abcam), rabbit anti-SOX9 antibody (1:400, AB5535, Millipore), and rabbit anti-Oxytocin antibody (1:400, ab212193, Abcam) as the primary antibody at 4°C overnight, and then incubated with Alexa Fluor 488 goat anti-mouse IgG (1:1000, A21202, Invitrogen, USA) or Alexa Fluor 594 donkey anti-rabbit IgG (1:1000, A21207, Invitrogen, USA) at 37°C for 1 h. All images were captured with laser confocal scanning microscopy (Leica TCS SP5).

### Papanicolaou staining of sperm

Epididymides of ZIKV-infected or control mice at 56 dpi were collected. After being clipped on the tail, epididymides were placed in PBS and incubated at 37°C for 20 min. The 50 µL of supernatant was placed on dry glass slides. After air-drying naturally, slides were first immersed in 95% ethanol for 10 min; then sequentially immersed in 85% ethanol, 80% ethanol, 70% ethanol, and purified water; and stained with hematoxylin. After differentiation by hydrochloric acid ethanol, the slides were sequentially stained with G-6 orange and EA-50. After being re-immersed in alcohol and xylene, slides were mounted by synthetic resin.

### Library preparation and RNA sequencing

RNA sequencing of the hypothalamus and testes harvested from ZIKV-infected or control mice at 10 and 56 dpi was commercially conducted (LC-Bio Technology Co., Ltd., Hangzhou, China). Three to four individuals were in each group. In brief, after extraction of total RNA (Trizol reagent, ThermoFisher, 15596018), mRNA was purified by Dynabeads Oligo (dT) (Thermo Fisher, CA, USA). The mRNA was fragmented by Magnesium RNA Fragmentation Module (NEB, cat.e6150, USA) to create cDNA libraries. A real-time PCR system was used to quantify and qualify the sample libraries. Finally, the cDNA libraries were sequenced using Illumina Novaseq 6000 (Illumina, Inc., USA).

### Bioinformatics analysis

To get high-quality clean reads, we further filtered reads using Cutadapt v1.9. For reads mapping, the reference genome (GRCm39) was downloaded from the Ensembl website (ftp://ftp.ensembl.org/pub/current_gtf). We aligned reads of all samples to the GRCm39 reference genome using HISAT2 v2.2.1 (48). StringTie and Ballgown were used to estimate the expression levels of all genes and transcripts and calculate the FPKM (fragment per kilobase of transcript per million mapped reads) value (49). Differentially expressed gene analysis between two groups and principal component analysis (PCA) were performed by DESeq2 v1.34.0 (50). Genes with adjusted *P*-value < 0.05 and |Log2 fold change| ≥ 1 were considered as DEGs. Gene ontology (GO) enrichment analysis, KEGG pathways enrichment analysis, and GSEA were conducted by clusterProfiler v4.2.2 (51). Graphics drawing was conducted by the ggplot2 v3.4.0 and ComplexHeatmap v2.13.1.

### Three-chamber task

The social behavior and social novelty preference of mice were examined by a three-chamber task (52). The apparatus which consisted of a clear rectangular plastic box (75 × 30 cm) was divided into three equal compartments (25 × 30 cm) by plastic dividers with an opening. Test mice were allowed to habituate in the middle chamber for 5 min when openings were closed by removable doors. The doors were then opened and mice were allowed to explore the entire box for 5 min. The social test consisted of 5 min where one of the side chambers was randomly selected as the social side and the other as the non-social side. In the social chamber, an 8-week-old male mouse (Stranger 1) was placed under a wire cup. The non-social side consisted of an empty cup. The social novelty preference test consisted of 5 min where the previous non-social side was put into another 8-week-old male mouse (Stranger 2) under a wire cup. Mice were videotaped during the entire session. The index of social novelty preference is calculated by (Stranger 2 time − Stranger 1 time) / (Stranger 1 time +Stranger 2 time) ×100%.

The sexual preference of male mice was examined by a three-chamber task. The apparatus is consistent with the above experiment. The sexual preference test consisted of 5 min where one of the side chambers was the female mouse side and the other was the male mouse side. A new female mouse (8 weeks old) and a new male mouse (8 weeks old) were placed in the side chambers under wire cups, respectively. Mice were videotaped during the entire session.

### Open field test

The open field test was carried out to evaluate the motor ability and autonomous exploration (53). The apparatus is a well-lit transparent square cage (50 × 50 cm) equipped with a black floor. Each mouse was placed in the corner of the cage. The total distance traveled (m), total freezing episodes, total time freezing(s), time spent in the center zone (40 × 40 cm), time spent in the limb zone, and maximum speed (m/s) were measured for 5 min by the ANY-maze system.

### Statistical analysis

R v4.1.2 and GraphPad Prism v7.0 were used for statistical analysis. The quantitative data between two groups with normal distributions were analyzed by Student’s *t*-test. All results in this study were presented as the mean ± standard deviation (SD) from at least three repeats. *P* < 0.05 (*), *P* < 0.01 (**), and *P* < 0.001 (***) was considered significant difference between the two groups.

## Data Availability

The raw sequencing data were deposited at the NCBI Sequence Read Archive (SRA) under the BioProject accession number PRJNA994603.
